# Predicting patient deterioration with physiological data using AI: systematic review protocol

**DOI:** 10.1136/bmjhci-2024-101417

**Published:** 2025-08-05

**Authors:** Lynsey Threlfall, Cen Cong, Victoria Riccalton, Edward Meinert, Chris Plummer

**Affiliations:** 1 Newcastle Upon Tyne Hospitals NHS Foundation Trust, Newcastle upon Tyne, UK; 2 NIHR Newcastle Biomedical Research Centre, Newcastle upon Tyne, UK; 3 Translational and Clinical Research Institute, Newcastle University, Newcastle upon Tyne, UK; 4 Department of Primary Care and Public Health, Imperial College London, London, UK

**Keywords:** Artificial intelligence, Machine Learning, Hospital Records, Emergency Service, Hospital

## Abstract

**Introduction:**

The second iteration of the National Early Warning Score has been adopted widely within the UK and internationally. It uses routinely collected physiological measurements to standardise the assessment and response to acute illness. Its use is associated with reduced mortality but has limited positive and negative predictive accuracy. There is a growing body of research demonstrating the effectiveness of artificial intelligence (AI) in predicting clinical deterioration, but there is limited evidence to show which aspect of AI is best suited to this task. This systematic review aims to establish which AI or machine learning algorithm is best suited to analysing physiological data sets to predict patient deterioration in a hospital setting.

**Methods and analysis:**

A systematic review will be conducted in accordance with the PRISMA (Preferred Reporting Items for Systematic Reviews and Meta-Analysis) and the PICOS (Population, Intervention, Comparator, Outcome and Study) frameworks. Eight databases (PubMed, Embase, CINAHL, Cochrane Library, Web of Science, Scopus, IEEE Xplore and ACM Digital Library) will be used to search for studies published from 2007 to the present that meet the inclusion criteria. Two reviewers will screen the studies identified and extract data independently, with any discrepancies resolved by discussion. The review is expected to be completed by January 2026, and the results will be presented in publication by June 2026.

**Ethics and dissemination:**

Ethical approval is not required as data will be obtained from published sources. Findings from this study will be disseminated via publication in a peer-reviewed journal.

**WHAT IS ALREADY KNOWN ON THIS TOPIC**

- Artificial intelligence (AI) has shown promise in predicting clinical deterioration, but guidance on selecting models suitable for hospital data structures is lacking.

**WHAT THIS STUDY ADDS**

- This review will identify the most accurate algorithms for predicting clinical deterioration using patients’ physiological data.

**HOW THIS STUDY MIGHT AFFECT RESEARCH, PRACTICE OR POLICY**

- It provides evidence to guide model selection, improving early detection and supporting AI integration in healthcare systems.

## Introduction

 Patient observation has been an integral part of clinical practice since antiquity. Florence Nightingale strongly advocated structured patient observations in the second half of the 19th century,^1^ and healthcare professionals’ systematic recording of vital signs in the UK was formalised from the 1950s.^2^ These repeated sets of physiological measurements are used to assess the severity of illness, and in 2012 the Royal College of Physicians introduced the National Early Warning Score (NEWS) to create a standardised approach to recognise and respond to patient deterioration.^3 4^ This was updated in 2017 as NEWS2 with changes including a dedicated section for patients with hypercapnic respiratory failure.^5^ NEWS and NEWS2 have been shown to reduce mortality,^6 7^ but have limited positive and negative predictive accuracy.^3 8 9^ A recent study showed that at the clinically significant 5-point threshold, NEWS2 has a sensitivity of 72%, a specificity of 86% and a number needed to evaluate of 8.2 for the composite of cardiac arrest, unplanned critical care admission or death within 24 hours of the observation.^10^

Since then, digital tools to record observations (e-Observations or eObs) have been adopted widely. NEWS2 alone is now used by 100% of ambulance trusts and 76% of acute trusts across England, UK.^11^ The wide adoption of digital tools provides an opportunity to use artificial intelligence (AI) tools to interrogate these routinely collected data and improve the predictive accuracy of NEWS2. Several studies have demonstrated the capability of machine learning (ML) models in predicting clinical deterioration, particularly in emergency departments.^12^,^14^ By using ML and other AI tools to monitor and interpret physiological data, it is possible to include a wider set of variables than humans would have in a healthcare setting. Incorporating additional variables derived from electronic health records was beneficial in improving the predictive accuracy of the scoring system than using just vital signs.^15 16^ Our recent scoping review evaluated potential additional variables that may be used to improve the predictive accuracy of NEWS2.^10^ This systematic review investigates the AI approach that would be most appropriate for interrogating these data. We also aim to establish whether the same AI approach would be suitable for additional variables such as age, comorbidity and prescribed medications to provide a more personalised NEWS. While there are a few existing systematic reviews about ML models for the prediction of clinical deterioration, they focused on the barriers to the implementation of existing models in hospital settings or the evaluation of model performance.^17 18^ What still needs to be added to the literature is how to choose an ML-based model that is accurate and suitable for predicting disease deterioration with the current patient data structure in hospitals. This systematic review aims to contribute to the existing body of evidence and provide a reference to promote the implementation of ML-based models in hospital settings.

## Methods

The Preferred Reporting Items for Systematic Reviews and Meta-Analyses (PRISMA; see online supplemental appendix A for the PRISMA checklist)^19^ and PICOS (Population, Intervention, Comparator, Outcome and Studies) frameworks^20^ were used to build the search strategy (table 1) and provide a framework for the review.

**Table 1:** PICOS framework

Population	Any adult inpatient over the age of 18, who has routinely collected physiological observations recorded, regardless of demographic background or clinical presentation.
Intervention	Studies evaluating machine learning or artificial intelligence types that are used to evaluate physiological data sets and predict deterioration.
Comparison	Not indicated.
Outcome	Deterioration is defined as increases in early warning score, unplanned intensive care admissions, cardiac arrest and death.
Study types	All study types detailing the creation or evaluation of a machine learning or artificial intelligence algorithm applied to detect the physiological deterioration of inpatients will be included. If there is no access to full text, the study will be excluded,

### Search strategy

Six databases will be searched to identify literature for screening: PubMed, Embase, CINAHL, Cochrane Library, Web of Science and Scopus. IEEE Xplore and ACM Digital Library will also be searched to ensure the inclusion of grey literature pertinent to the review question. Keywords and MeSH (Medical Subject Headings) terms were categorised into three key areas to direct and structure the search (table 2). A sample search in Embase is included in online supplemental appendix B as an example.

**Table 2:** MeSH terms and keywords used for literature search

Category	MeSH	Keywords (in title or abstract)
Artificial intelligence	Artificial intelligence	“Artificial Intelligence” OR “AI” OR “A.I.” or “machine learning” or “ML”
Physiological observations	Monitoring, physiological	“early warning score” OR “early warning scores” OR “early warning systems” OR “patient monitoring” OR “vital sign monitoring” OR “vital signs” OR “vital signs monitoring”
Clinical deterioration	Deterioration, clinical	“clinical deterioration” OR “patient deterioration” OR “detection” OR “prediction” OR “deterioration”

MeSH, Medical Subject Headings.

### Inclusion criteria

All studies that examine the use of an AI or ML approach to analysing physiological data sets or predicting deterioration within a hospital setting will be eligible for inclusion. Grey literature will also be eligible for inclusion in the study as long as it discusses the use of an AI approach to detecting or predicting clinical deterioration using vital signs within a hospital setting.

### Exclusion criteria

Abstracts with no full text or studies not published in English will not be eligible for inclusion due to the language capabilities of the authors. Papers published prior to 2007 will also not be eligible. This systematic review is part of a wider programme of work to create a tool that can be used in digital healthcare records using a smartphone and a version on paper charts.

Studies that look at AI or ML to interpret other aspects of healthcare provision, such as the screening of patients for procedures or medical images, will not be suitable for inclusion.

### Screening and article selection

The citation management software EndNote V.21 (Clarivate) was used for storing references, removal of duplicated references and keyword-based screening. After that, a set of references will be uploaded to Rayyan (https://www.rayyan.ai/) for screening. The screening based on titles and abstracts will be shared between two and four authors, and full-text screening will be performed by two authors. Any conflicts in screening will be resolved through discussion between two reviewers. A third reviewer will be involved to address any agreement that cannot be reached between the two reviewers.

### Data extraction

Two independent reviewers will extract data from the included studies based on a predetermined data extraction form (see box 1). Conflicts in extraction will be resolved by discussion between reviewers. The Checklist for Critical Appraisal and Data Extraction for Systematic Reviews of Prediction Modelling Studies (CHARMS) framework^21^ was used to guide data extraction and comparison of methodological characteristics across studies.

Box 1 **Article information and data extraction**

**Literature**

- Author.
- Title.
- Year of publication.
- Country of study.
- Sample size.
- Study type.
- Population.
- Intended scope of study.

**Deterioration tools**

- Tool used in the study (eg, NEWS/Queensland Adult Deterioration Detection System (Q-ADDS)/Sequential Organ Failure Assessment (SOFA)).
- Type of artificial intelligence technique used (eg, machine learning and deep learning).
- Type of algorithm used (eg, supervised and unsupervised machine learning).
- Performance of algorithm.
- Patient outcomes.
- Model validation method.
- Time span of prediction.
- Intended moment of using the model.
- Comparator (if any).
- Performance of comparator.
- Types of physiological data captured.
- Impact on patient outcome.
- Impact on service delivery.

### Data analysis and synthesis

Data extracted based on the above-predetermined outcomes will be analysed descriptively to provide an understanding of the type and performance of common AI algorithms used in clinical deterioration tools and the types of physiological data used for prediction. The performance of each AI algorithm will be evaluated based on its accuracy, precision, recall, F1-score, area under the curve and receiver-operating characteristic curve. The quality of each included study will be assessed and compared based on the CHARMS framework. Depending on the quantity and quality of data extracted, a meta-synthesis will also be performed to understand the impact of various AI algorithms on patient outcomes.

## Supplementary material

10.1136/bmjhci-2024-101417 online supplemental file 1

10.1136/bmjhci-2024-101417 online supplemental file 2
