# Supplementary material for: Predicting patient deterioration with physiological data using AI: systematic review protocol
Source: BMJ Health Care Inform. 2025 Aug 5;32(1):e101417. doi: 10.1136/bmjhci-2024-101417 (PMC12336570; doi:10.1136/bmjhci-2024-101417)
Supplement: online supplemental file 2 [file bmjhci-32-1-s002.docx]

**Appendix B. Sample Search**

| Database | Search String | Results |
| --- | --- | --- |
| Embase (1974-2024 database) | ("Artificial intelligence" OR "AI" OR "A.I." OR "machine learning") AND ("Physiological observations" OR "physiological monitoring" OR "vital signs") AND ("Clinical deterioration" OR "deteriorating patient" OR "patient deterioration" OR deterioration*) | 108 |
